# The Automatic Algorithm of the Auto-CPAP Device as a Tool for the Assessment of the Treatment Efficacy of CPAP in Patients with Moderate and Severe Obstructive Sleep Apnea Syndrome

**DOI:** 10.3390/life12091357

**Published:** 2022-08-31

**Authors:** Beata Brajer-Luftmann, Tomasz Trafas, Marcin Mardas, Marta Stelmach-Mardas, Halina Batura-Gabryel, Tomasz Piorunek

**Affiliations:** 1 Department of Pulmonology, Allergology and Pulmonary Oncology, Poznan University of Medical Sciences, Szamarzewskiego 84 Street, 60-569 Poznan, Poland; 2 Department of Ginecological Oncology, Institute of Oncology, Poznan University of Medical Sciences, Szamarzewskiego 84 Street, 61-569 Poznan, Poland; 3 Department of Treatment of Obesity, Metabolic Disorders and Clinical Dietetics, Poznan University of Medical Sciences, Szamarzewskiego 84 Street, 61-569 Poznan, Poland

**Keywords:** OSAS, polysomnography, polygraphy, CPAP

## Abstract

Obstructive sleep apnea syndrome (OSAS) is a common sleep-related breathing disorder where precise treatment assessment is of high importance. We aimed to validate an automatic algorithm of the auto-CPAP device and reveal polygraph usefulness in the OSAS diagnosis and treatment of outpatients. One hundred patients with moderate OSAS, severe OSAS, and excessive daytime sleepiness qualified for CPAP treatment were included. The study was conducted in three stages. The first stage included a minimum 6-hour polysomnographic examination to select moderate and severe OSAS. The second stage involved an auto-CPAP treatment lasting at least 4 h with simultaneous polygraph recording. The third stage was a titration of at least 4 h with auto-CPAP. The Apnea–Hypopnea Index (AHI) and oxygen desaturation index (ODI) were calculated under auto-CPAP treatment, simultaneously using polygraph (stage two), and as a result of treatment with auto-CPAP (stage three). The mean AHI was 40.0 ± 20.9 for OSAS. Auto-CPAP treatment was effective in 97.5%. The mean residual AHI was 8.6 ± 4.8; there was no significant difference between the AHI CPAP, and the AHI polygraph values were assessed with an accuracy of 3.94/h. The sensitivity and specificity of calculated cut point 8.2 event/hour were: 55% and 82%, respectively. The calculated AUC for the AHI CPAP parameter was 0.633. Presented data confirmed that the automatic algorithm of auto-CPAP is a good tool for the assessment of the treatment efficacy of CPAP in patients, i.e., home setting, with a moderate or severe stage OSAS-presented high sleepiness.

## 1. Introduction

Obstructive sleep apnea syndrome (OSAS) is a common, but often unrecognized, disorder [1]. It was estimated that nearly one billion adults aged 30–69 years worldwide could have obstructive sleep apnea. The number of people with moderate to severe obstructive sleep apnea, for which treatment is generally recommended, is estimated to be almost 425 million [2]. It is a disorder characterized by the repeated complete (apnea) or partial (hypopnea) narrowing of the upper respiratory tract with the work of the respiratory muscles preserved [3,4,5]. The consequence of OSAS is the deterioration of blood oxygenation and frequent (usually unconscious) awakenings, leading to defragmentation of sleep and excessive daytime sleepiness, which leads to an increased prevalence of cardiovascular diseases in patients with OSAS [3,4,5,6,7,8]. In our previous study, we confirmed that lifestyle modification resulting in the reduction of one unit of body mass index (BMI) gives meaningful and positive changes in selected cardio-metabolic risk factors, such as total cholesterol (TC), triglycerides (TG), fasting insulin, and blood pressure (BP), in OSAS patients [9].

However, the gold standard treatment for OSAS is continuous positive airway pressure (CPAP) therapy [10]. Titration with the CPAP device is a method that relies on the continuous generating of overpressure in the upper airways to prevent their collapse [11]. The standard is the selection of therapeutic pressure through a simultaneous polysomnographic recording during which the patient is treated with a CPAP device. The method of selecting pressure depends on the type and frequency of respiratory disorders [12]. CPAP treatment is recommended in severe disease with Apnea–Hypopnea Index (AHI) > thirty, moderate disease with AHI > fifteen and severe daytime sleepiness assessed on Epworth Sleepiness Scale (ESS) ≥ eleven points or cardiovascular complications, and mild disease of five ≤ AHI ≤ fifteen with severe daytime sleepiness [4,13]. According to the American Academy of Sleep Medicine (AASM) guidelines, the attempted therapy should take place in a sleep laboratory [14]. In Poland and also in poor-income countries, the availability of specialized laboratories is still insufficient, both at the stage of OSAS diagnosis and treatment implementation [15]. The situation is made worse by the lack of outpatient procedures. There seems to be a justified need for research aimed at demonstrating the usefulness of outpatient devices in the diagnosis and treatment of OSAS.

The presented work is an attempt to validate the automatic algorithm of the auto-CPAP device to reduce breathing disorders during sleep in patients with OSAS. In this way, the authors tried to check that the automatic algorithm of the auto-CPAP treatment may be enough for OSAS treatment effectiveness assessment in a situation of limited access to polysomnography (PSG), especially in primary care.

## 2. Materials and Methods

### 2.1. Study Design

This is a prospective observational study. Patients were recruited between July 2019 and June 2020 at the Department of Pulmonology, Allergology, and Pulmonary Oncology, Poznan University of Medical Sciences (Poland). All included patients were diagnosed and treated in the University Sleep Laboratory. All procedures were conducted to good laboratory and diagnostic practices.

The study protocol was approved by the Bioethical Committee at Poznan University of Medical Science 7 March 2019 (No: 339/19). All enrolled participants provided written informed consent. The study was conducted in accordance with the Helsinki Declaration.

### 2.2. Study Population

One hundred adult patients (fifty men and fifty women) were included in the study according to the following criteria: age over 18, moderate and severe OSAS diagnosed, and associated excessive daytime sleepiness qualified for treatment with CPAP. The exclusion criteria were as follow: the lack of excessive daytime sleepiness; impaired patency of the upper respiratory tract resulting in reduced effectiveness of positive pressure therapy—indications for ear, nose, and throat (ENT) intervention, taking hypnotic, and/or sedatives.

Excessive daytime sleepiness was assessed with the Epworth Sleepiness Scale (ESS) and defined as achieving ≥ 11 points [16]. The diagnosis of OSAS was performed according to AASM recommendations [14,17] using a polysomnography machine (Alice 6, Philips, PA, USA, 2017).

### 2.3. Auto-CPAP Device Validation

The study was carried out in three independent stages, performed during a stay in the Sleep Laboratory for 2 days.

The first stage included a minimum 6-hour polysomnographic examination (Polysomnograph Alice 6, Murrysville, PA, USA, 2017) with a particular assessment of the following parameters: AHI, oxygen desaturation index (ODI), average saturation, and stages of sleep (NREM-N1, N2, N3, and REM). The analysis was performed with the Sleepware G3 software (Respironics, Murrysville, PA, USA, 2019).

The second stage involved treatment with an auto-CPAP lasting at least 4 h with simultaneous polygraphy (PG) recording. In this stage, the Alice Night One polygraph (Philips, PA, USA, 2017) and auto-CPAP Dreamstation (Philips, PA, USA, 2017) were used with a built-in automatic algorithm to maintain the patency of the upper respiratory tract. The values of AHI, ODI, average saturation, therapeutic pressure representing the 90th percentile of pressures generated during therapy, and average pressure generated with the auto-CPAP device were recorded. To distinguish the parameters used in analyses for this study the AHI indicator from the polygraph’s test has been modified into an abbreviation AHI PG. The desaturation index from the PG study was designated ODI PG. The analysis was performed with Sleepware software (Respironics, PA, USA, 2019).

The third stage of the study was a titration of at least 4 h with the auto-CPAP apparatus taking into account the following parameters: AHI residual respiratory disorder index, 90th percentile therapeutic pressure generated over time pressure therapy, and the average pressure generated during treatment with the auto-CPAP device. Each patient was provided with a comfortable and tight mask that allowed free breathing. To distinguish the parameters used in the analyses for this study, the AHI indicator from auto-CPAP titration was modified to the abbreviation AHI CPAP. The Encore Basic (Respironics, PA, USA, 2019) software was provided by the auto-CPAP manufacturer. According to the manufacturer’s information, auto-CPAP responds to respiratory events in accordance with the principles of manual titration.

### 2.4. Statistical Analysis

A population of 105 subjects was required to show differences with type I error stated as alpha 0.05 and with the power of 95%. The data were expressed as mean, median, and standard deviation. The normality of the distribution was checked by the Shapiro–Wilk test. The Mann–Whitney test was used to analyze the differences between the variables from the diagnostic stage and auto-CPAP therapy and to describe the differences before and after medical intervention. Using the Spearman’s R correlation the relationship between AHI and ODI indices from the therapeutic process was demonstrated. The validation of the automatic algorithm of the auto-CPAP apparatus was made using the Bland– Altman chart and a mountain plot. A *p*-value < 0.05 defined statistically significant differences. All calculations were performed with the use of Statistica 10 software (TIBICO Software Inc., Palo Alto, CA, USA, 2017).

## 3. Results

**Figure 1:**
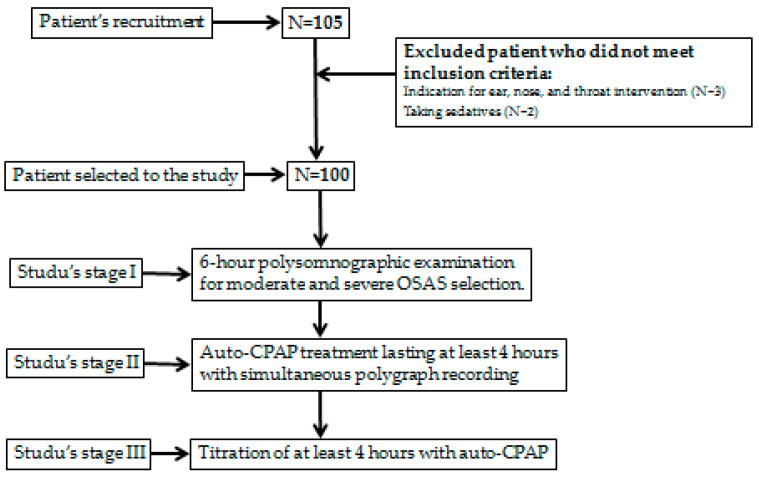
The patient sample selection.

One hundred five adult patients were recruited for the study. Five subjects did not meet inclusion criteria (indications for (ENT) intervention—three patients, and taking sedatives—two patients)—Figure 1.

The study included 100 (50% men) patients diagnosed with at least moderate OSAS and associated excessive daytime sleepiness (ESS ≥ 11 points) who qualified for treatment with CPAP air prosthesis. The basic features of the study population and the clinical characteristics of patients with moderate and severe OSAS are presented in Table 1.

**Figure 2:**
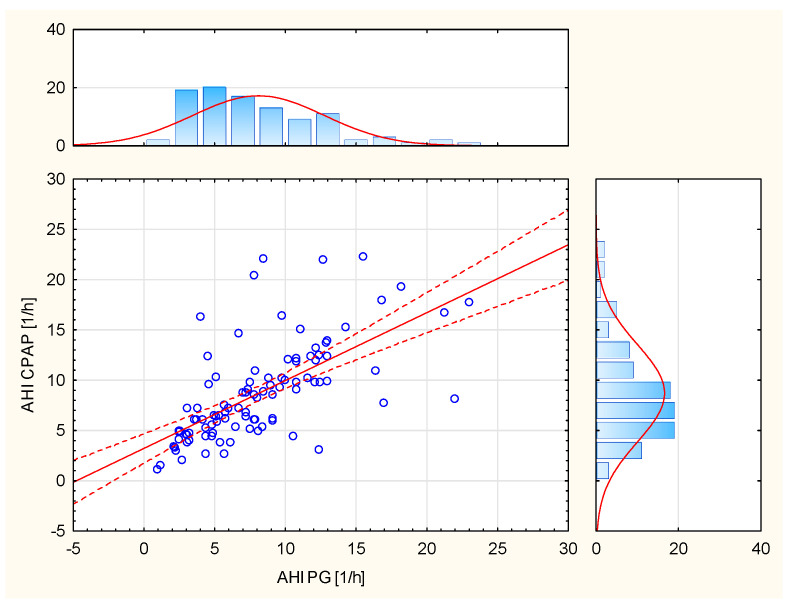
Relationship between AHI CPAP and AHI PG.

The results of the Mann–Whitney U Test (*p* = 0.5126), with the adopted significance level (α = 0.05), indicate statistically significant differences between the distributions of variables from the diagnostic stage (PSG) and after the CPAP treatment process. On the other hand, the results of the Mann–Whitney U Test (*p* < 0.0001) indicate no significant differences between the AHI values derived from CPAP and polygraph (PG). Spearman’s rank correlation (R = 0.7193, *p* < 0.0001) shows a significant relationship between AHI CPAP and AHI PG (Figure 2).

In order to validate the operation of the automatic algorithm of the CPAP device, statistical methods were used to compare the AHI residual index values from the titration during CPAP titration and the AHI and ODI values from the manual interpretation of the polygraphy (AHI PG and ODI PG).

A significant correlation was demonstrated between the AHI parameters analyzed by CPAP and scored in polygraphy (Figure 2). However, a significant relationship was demonstrated between the parameters of AHI analyzed by CPAP and the ODI scored in polygraphy (Table 2).

**Figure 3:**
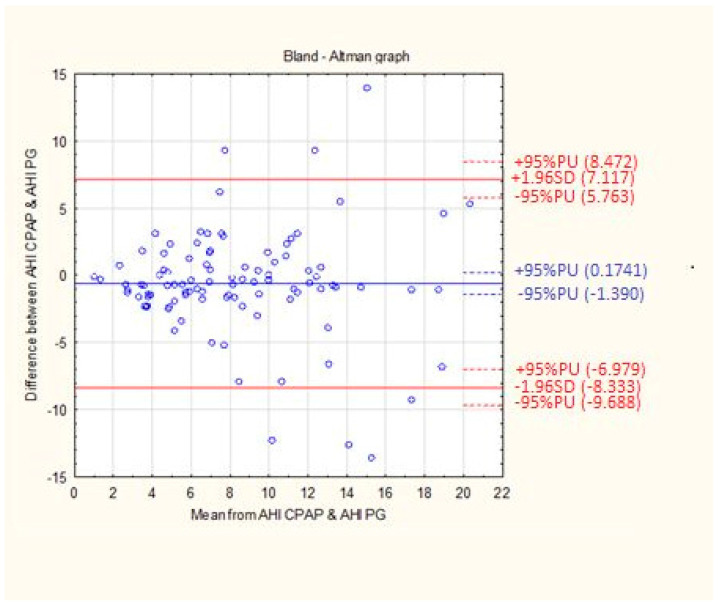
The mean difference between the AHI CPAP and AHI PG. The data are presented using Bland-Altman graph.

Differences between the results of the residual respiratory disorder index obtained from individual patients by means of apparatus titration auto-CPAP and the results from the Alice Night One polygraph were compared with the mean value of the results obtained from both test methods. The solid blue line represents the error systematic and the dashed red lines indicate the 95% compliance limits (Figure 3).

The mean difference between the AHI CPAP and AHI PG values was 0.6080 l/h with an accuracy of 3.94 1/h (Table 3).

**Figure 4:**
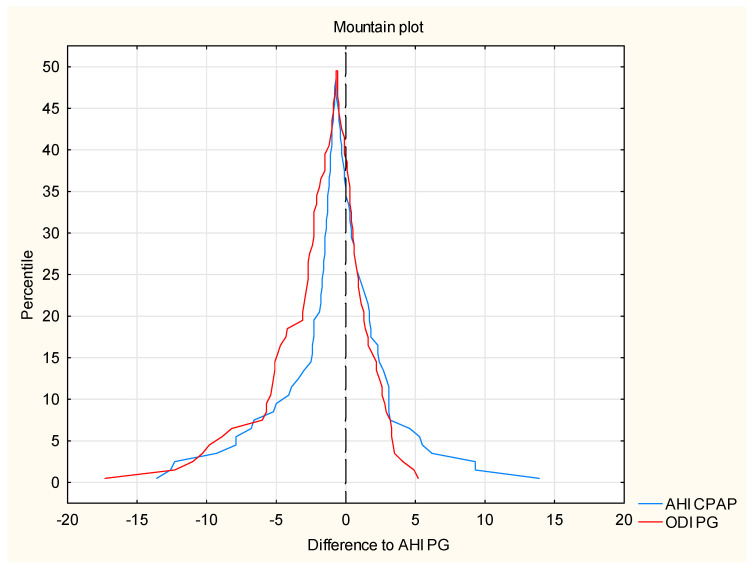
The correlation between the AHI PSG and ODI PSG values (mountain plot).

To confirm the identity of both methods, a mountain plot was made, which is a graphical representation of the differences in the values of AHI CPAP residual indices and ODI PG relative to the expected value. A strong positive correlation between the AHI PSG and ODI PSG values (Figure 4) was used for the preparation mountain plot.

By analogy, the value of the AHI PG index has been established as the expected value for the residual respiratory AHI CPAP disturbance indices and ODI PG. The chart is created by determining the percentiles for the ranked ones ascending differences between the AHI CPAP and ODI PG values relative to the value expected AHI PG. A slight shift in the maximum peaks is noticeable as it curves from the zero point, which proves the high compliance with the expected value. The shape of the curves confirms a little discrepancy in the determination of the residual index breathing disorders between both methods.

**Figure 5:**
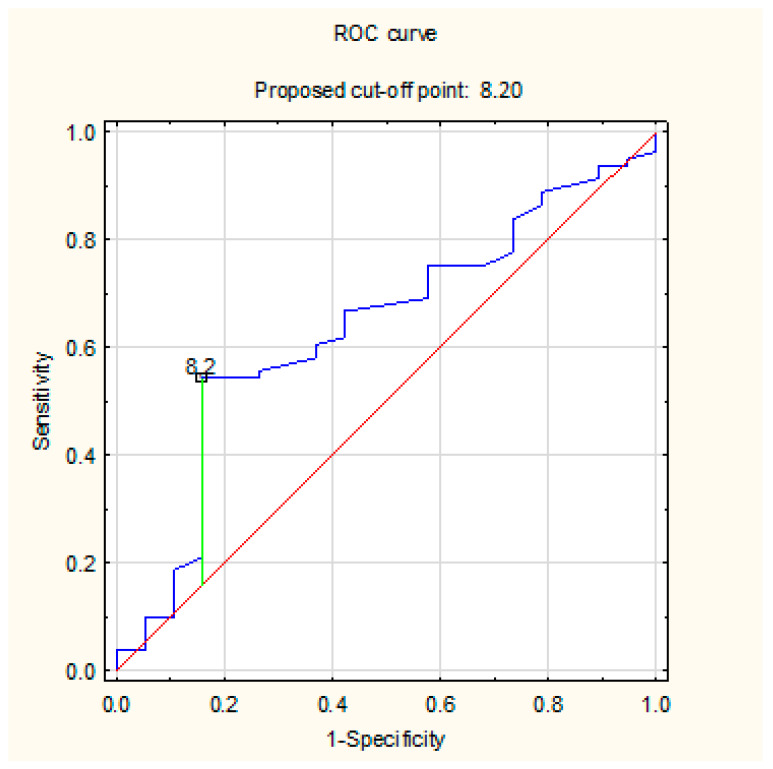
The sensitivity and specificity of the AHI parameter PSG and the cut-off point above which the value of the residual index AHI CPAP is most consistent with the real value using ROC curve.

The determined ROC curves show the sensitivity and specificity of the AHI parameter PSG (Figure 5) and the cut-off point above which the value of the residual index AHI CPAP is most consistent with the real value. The sensitivity and specificity of the calculated cut point 8.2 1/h are 55% and 82%, respectively. The calculated AUC for the AHI CPAP parameter is 0.633.

## 4. Discussion

The current study is an attempt to validate the automatic algorithm of the auto-CPAP device in the reduction in breathing disorders during OSAS treatment. According to the current AASM guidelines [17], therapy with the CPAP device should be monitored using a polysomnographic device. The insufficient number of polysomnographic laboratories in Poland (currently OSAS diagnostics can be performed in 57 canters), the long waiting time for the examination, and the lack of reimbursed diagnostic procedures performed on an outpatient basis were the reasons for the author to look for a reliable diagnostic and therapeutic method that could improve the existing situation. The possibility of titration with an auto-CPAP device was analyzed without needing to use devices to supervise the operation of an air prosthesis and to evaluate the value of residual AHI. The obtained results indicate a 97.5% effectiveness of AHI reduction as a result of auto-CPAP therapy. The results of the current study suggest that in the case of limited access to PSG, an automatic algorithm of the auto-CPAP seems a sufficient tool to assess the effectiveness of OSAS treatment.

Objective assessment of this issue required a careful selection of patients participating in this project. Corral et al. [18] showed a high agreement of the AHI respiratory disturbance index values obtained from type one and type three devices, according to the AASM guidelines [19]. It has been shown that the sensitivity of the polygraph increases with the value of the AHI respiratory disturbance index [20]. Taking into account the effectiveness of polygraph diagnostics and the poor availability of polysomnography, other authors decided to use a polygraph to evaluate the titration of the auto-CPAP apparatus [21,22].

The current study results revealed a high effectiveness of CPAP treatment using polygraphy and automatic CPAP algorithm. Similar effectiveness of positive pressure therapy was demonstrated by Kotzian et al. [19], pointing to the groups of patients for whom a correction of the therapeutic pressure value is required to enable an effective reduction in AHI. Immediate reduction in daytime sleepiness was confirmed in the majority of patients, distinguishing at the same time a group for which the effect of one night was not sufficient [19]. Similar observations were made by Djonlagic et al. [20]. The authors of this study validated the effectiveness of reducing respiratory disturbances by an automatic algorithm for recognizing respiratory events in which the auto-CPAP device is equipped. Some of the performed statistical analyses showed high compliance of the residual index of respiratory disorders from auto-CPAP and the monitoring device polygraph. Spearman’s rank correlation showed a significant relationship between the AHI CPAP and AHI PG values and a clear relationship between AHI CPAP and ODI PG. Gagnadoux et al. [23] confirmed these results. The Passing–Bablok regression analysis carried out by Gagnadoux et al. [23] showed the identity of both research methods. According to the interpretation of the statistical method used, auto-CPAP titration, without the use of surveillance equipment, can be used to determine the residual AHI value, which is a cheaper and simpler method of initial treatment.

Our study showed that titration performed only with the auto-CPAP algorithm is sufficient for the proper course of OSAS therapy in patients with moderate and severe disease, excessive daytime sleepiness, and no indications of ENT treatment. The results of research works published by Corral [18], Botokeky [24], and Nigro [25] confirm these observations. Auto-CPAP titration after the first month of treatment is more effective than manual inpatient titration in patients requiring positive pressure therapy and without serious comorbidities. This results in better adherence to the recommendations for CPAP therapy, a noticeable increase in the number of patients using long-term CPAP, and a reduced percentage of patients discontinuing treatment [20]. Observation of the therapy at home by partners has a positive effect on patient’s compliance with treatment with positive blood pressure [22,24]. On the other hand, over the years, psychological measures of behavior change constructs have been increasingly recognized as the most consistent predictors of CPAP adherence and, as a result, the most successful interventions to optimize adherence have been behavioral. Combining theory-based behavioral approaches with telemedicine technology could be the answer to increasing CPAP adherence rates in the real world, although randomized trials are still needed and socioeconomic barriers to telemedicine will need to be addressed to promote health equity [26].

The Bland–Altman difference diagram used in the current study showed a high agreement of the obtained residual AHI values from both methods. The mean difference in the obtained measurements was 0.6080 events per hour. One of the criteria for enrolling patients in the study was the diagnosis of excessive daytime sleepiness. Effective therapy with positive pressure, objectively confirmed in the measurements carried out, also allows the subjective feeling of the effects of treatment, among others in the form of relief from sleepiness. Noseda et al. [27], in his work, showed that in patients with excessive daytime sleepiness, titration with auto-CPAP is as effective as manual titration with polysomnography and CPAP prosthesis, and the obtained therapeutic pressures do not differ significantly. In our study, we confirmed the equivalence of both methods in determining the residual AHI, which was presented graphically by means of a slope chart. The shape of the obtained graph, as well as a slight shift of the peaks in relation to the zero value, indicate a high compatibility of both methods. The presented study indicates no statistically significant differences between the distributions of AHI CPAP and AHI PG. The results of our analysis are confirmed in the previously conducted research by Li et al. [28]. In purpose to show statistical differences between the values of the parameters AHI, ODI, mean saturation, and the lowest saturation, this study was supplemented with a non-parametric analysis of variance test, which showed that the variables from two study stages do not belong to one population. Herkenrath et al. [29] showed similar conclusions, showing statistically significant differences between the medians of the AHI index during diagnosis and treatment with the auto-CPAP device.

## 5. Limitations

This study has a few limitations. It was conducted only in one center. The study group consisted of patients with moderate to severe OSAS and high sleepiness, creating a relatively small sample size. This is a limitation of the possibility of extrapolating the obtained results to the general population of patients with OSAS. In the patients included in the study, we noticed problems with the proper subjective assessment of sleepiness. Therefore, it was eliminated mainly by collecting an interview from accompanying persons—sleep partners. A certain inconvenience of the study is the failure to obtain data distribution in accordance with the normal distribution, resulting, inter alia, from the non-random selection of patients and the numerical predominance of people with the severe stage of the disease.

## 6. Conclusions

In conclusion, our study results suggest that the automatic algorithm of the auto-CPAP device is a good tool for the assessment of the treatment efficacy of CPAP in patients with a moderate or severe stage OSAS-presented high sleepiness. This method of assessing the effectiveness of CPAP treatment can be used especially in primary care and in countries where access to PSG is still insufficient.

## Figures and Tables

**Table 1:** CPAP treatment results analyzed using auto-CPAP with polygraph.

Analyzed Variable	Mean	Minimum	Maximum	SD	Lower Quartile	Upper Quartile	Median	*p*-Value
AHI CPAP (1/h)	8.6	1.1	22.3	4.8	4.9	10.9	7.6	<0.0001
AHI PG (1/h)	8.3	1.0	23.0	4.6	4.5	10.8	7.5	<0.0001
ODI PG (1/h)	9.4	2.1	39.3	6.2	5.8	10.7	7.9	<0.0001
Mean saturation PG (%)	92.3	74.0	96.0	3.6	91.0	95.0	3.6	<0.0001
Lowest saturation PG (%)	83.7	58.0	93.0	6.5	81.0	88.5	6.5	<0.0001

Abbreviations: AHI CPAP, Apnea–Hypopnea Index analyzed by auto-CPAP; AHI PG, Apnea–Hypopnea Index scored by polygraph; ODI PG, oxygen desaturation index scored by polygraphy; PG, polygraphy.

**Table 2:** The relationship between AHI analyzed by CPAP and the ODI scored in polygraphy.

Analyzed Variable	R Spearman	*p*-Value
AHI CPAP and AHI PG	0.7193	<0.0001
AHI CPAP and ODI PG	0.4435	<0.0001

Abbreviations: AHI CPAP, Apnea–Hypopnea Index analyzed by auto-CPAP; AHI PG, Apnea–Hypopnea Index scored in polygraphy; ODI PG, oxygen desaturation index scored in polygraphy; PG, polygraphy.

**Table 3:** The mean difference between the AHI CPAP and AHI PG.

Analyzed Variable	Mean Difference	SD	Standard Error
Difference between AHI CPAP and AHI PG	0.6080	3.94	0.39

These data are presented using Bland–Altman analysis. Abbreviations: AHI CPAP, Apnea–Hypopnea Index analyzed by auto-CPAP; AHI PG, Apnea–Hypopnea Index scored in polygraphy.

## Data Availability

To obtain access to secondary data, please contact corresponding author.
